# Divergent memory responses driven by adenoviral vectors are impacted by epitope competition

**DOI:** 10.1002/eji.201948143

**Published:** 2019-05-29

**Authors:** Julia M Colston, Claire Hutchings, Senthil Chinnakannan, Andrew Highton, Christian Perez‐Shibayama, Burkhard Ludewig, Paul Klenerman

**Affiliations:** ^1^ Nuffield Department of Medicine Peter Medawar Building for Pathogen Research, University of Oxford Oxford UK; ^2^ Institute of Immunobiology Kantonsspital St. Gallen St. Gallen Switzerland

**Keywords:** adenoviral vectors, immunoproteasome, memory inflation, minigenes, LCMV

## Abstract

Adenoviral vectors induce robust epitope‐specific CD8^+^ T cell responses. Within the repertoire of responses generated both conventional memory evolution and the phenomenon of memory inflation are seen. The rules governing which epitopes inflate are not fully known, but may include a role for both antigen processing and competition. To investigate this, we looked at memory generated from vectors targeting the Gp33‐41 (KAVYNFATC/K9C) epitope from the gp of lymphocytic choriomeningitis virus (LCMV) in mice. This well‐described epitope has both the Gp33‐41 and Gp34‐41 epitopes embedded within it. Vaccination with a full‐length gp or a minigene Ad‐Gp33/K9C vector‐induced conventional memory responses against the immunodominant Gp33/K9C epitope but a strong inflationary response against the Gp34/A8C epitope. These responses showed sustained in vivo function, with complete protection against LCMV infectious challenge. Given the unexpected competition between epitopes seen in the minigene model, we further tested epitope competition using the full‐length Ad‐LacZ (β‐galactosidase) model. Generation of an Ad‐LacZ vector with a single amino acid disruption of the inflationary β‐gal_96‐103_/D8V epitope transformed the β‐gal_497‐504_/I8V epitope from conventional to inflationary memory. This work collectively demonstrates the importance of epitope competition within adenoviral vector inserts and is of relevance to future studies using adenoviral vectored immunogens.

## Introduction

T cell memory inflation is well described in the context of murine CMV (MCMV) infection 1. Certain epitope‐specific CD8^+^ T cell populations are noted to expand after an initial viral infection and remain dominant and functional over the life span of the host 1, 2, 3, 4, 5. We have previously described an adenoviral model of memory inflation, based upon a recombinant nonreplicating human adenovirus serotype 5 (AdHu5) with an insert of β‐galactosidase (β‐gal)/LacZ (Ad‐LacZ) 6. Within β‐gal, two Kb‐restricted epitopes have been identified, β‐gal_96‐103_/D8V (DAPIYTNV), which inflates, and β‐gal_497_‐_504_/I8V (ICPMYARV), which demonstrates conventional memory. The Ad‐LacZ model has been shown to replicate what is seen in MCMV memory inflation 6, 7.

Memory inflation has been previously described to depend upon the antigen‐processing context of the epitope. Inflationary epitopes tend to be immunoproteasome (specifically, low‐molecular mass protein‐7/LMP7)‐independent 6, 8. We reasoned that adenoviral vectors expressing epitopes as “minigenes” (requiring no further processing) may allow conversion of conventional/non‐inflationary epitopes to inflationary ones, with previous experiments validating this concept. Both I8V (Ad‐LacZ) and M45 (MCMV) are examples of immunoproteasome (LMP7)‐dependent epitopes that induce conventional responses when processed from their natural context but that can drive strong inflationary responses if the antigenic context is modified 9.

To look at this further, we developed a lymphocytic choriomeningitis virus (LCMV) minigene model. This includes a full‐length gp vector Ad‐Gp, and minigene vectors; Ad‐Gp33 (K9C:KAVYNFATC) and Ad‐Gp34 (A8C:AVYNFATC). We were surprised to find that the CD8^+^ T‐cell responses to all vectors were dominated by an inflationary population directed against the shorter Gp34/A8C epitope, compared to a conventional memory response against the Gp33/K9C epitope, suggesting a role for competition (with both epitopes showing LMP7‐independence 10). To assess this further, we directly tested the role of competition between epitopes within the Ad‐LacZ model. We designed a full‐length Ad‐LacZ vector with the β‐gal_96‐103_/D8V epitope disrupted (Ad‐disrupted D8V (LacZ)) using a single amino acid mutation in an anchor residue. This vector induced a dramatically altered, inflationary response from the β‐gal_497‐504_/I8V epitope, where normally a conventional response is seen. This work has implications for models of memory inflation and, translationally, immunodominance in response to adenoviral vector immunogens.

## Results and discussion

### Ad‐Gp and Ad‐Gp33 vectors generate an inflationary response, but only against the Gp34/A8C epitope

Initially we analyzed CD8^+^ T cell responses in mice immunized with the full‐length LCMV Gp (Ad‐Gp) vector. We tracked both Gp33 (KAVYNFATC/K9C) and Gp34 (AVYNFATC/A8C) tetramer^+^ CD8^+^ T cell responses in blood, following intravenous immunization into C57BL/6 mice. The Gp33/K9C tetramer^+^ populations displayed a conventional/non‐inflationary kinetic. In contrast, the Ad‐Gp immunization induced a robust Gp34/A8C tetramer‐specific CD8^+^ T cell response, with a sustained inflationary kinetic (Supporting Information Fig. 1).

**Figure 1:**
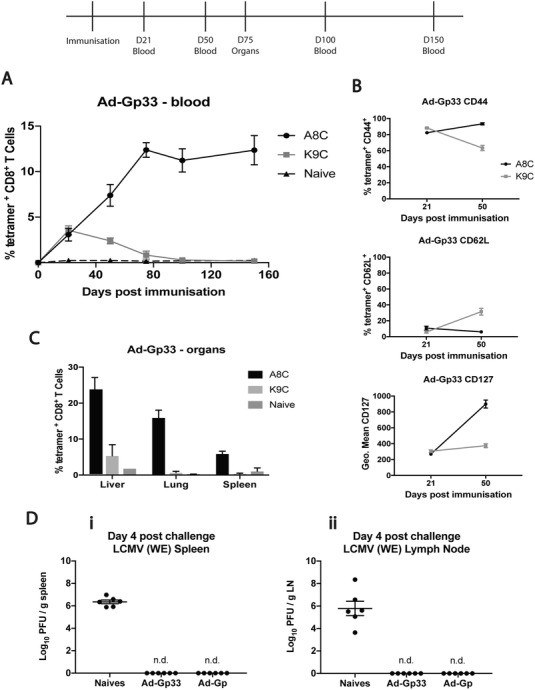
**(A)** Gp33/K9C and Gp34/A8C tetramer‐specific responses by flow cytometry in blood at time‐points 21, 50, 75, 100, and 150 post‐immunization with Ad‐Gp33 compared to naïve mice. Data pooled from three separate experiments up to day 50: *n* = 13, Day 75 onwards is one experiment (*n* = 4). Mean ± SEM shown. (**B)** A8C and K9C tetramer‐specific CD8^+^ T cells showing CD44, CD62L, and CD127 positivity by flow cytometry at day 21 and 50‐post immunization with Ad‐Gp33. Data shown from one experiment (*n* = 6), and representative of two independent experiments (*n* = 12). Mean ± SEM shown. (**C)** A8C and K9C tetramer‐specific responses by flow cytometry in organs at day 75‐post immunization with Ad‐Gp33, compared to naïve mice. Data shown from one experiment (*n* = 4), and representative of two independent experiments (*n* = 8). Mean ± SEM shown. (**D)** Demonstrates LCMV plaque assays performed on spleens (**i)** and lymph nodes (inguinal) (**ii)** at day 4‐post LCMV_WE_ challenge of C57BL/6 mice pre‐immunized (day 50) with Ad‐Gp33 and Ad‐Gp compared to naïve mice. Data are pooled from two separate experiments (*n* = 6). Mean ± SEM shown.

Based upon previous minigene work, we had expected the Ad‐Gp33 minigene to generate an inflationary response to the Gp33 epitope. However, the results following immunization showed the same divergent responses as seen in the Ad‐Gp construct. Gp33/K9C epitope‐specific CD8^+^ T cells showed conventional contraction and emergence of central memory pools without enrichment in peripheral organs. However, Gp34/A8C epitope‐specific CD8^+^ T cells showed memory inflation with a sustained peripheral kinetic over time accompanied by an effector memory phenotype and enrichment in organs (Fig. 1a–c). Overall these data demonstrate that two divergent memory responses can be generated from the same peptide immunogen in parallel, even when processing requirements are bypassed.

It was originally noted in studies of a recombinant MCMV expressing LCMV Gp33 at the N terminus of IE2 that inflation was seen against Gp34, and no sustained response observed against Gp33 11. In the setting of MCMV, there are numerous other endogenous epitopes that may be competing and additionally immune evasion genes that could influence peptide loading. However, in the current experiments, peptide processing is bypassed and only two epitopes are present. Furthermore, responses to both epitopes are equally well primed by the full‐length and the minigene vectors, but only the Gp34 response is sustained. The existence of two epitopes within Gp33‐41 has long been recognized, as is the immunodominance of these epitopes seen in natural infection 12. The importance of ER aminopeptidase 1 (ERAP1) and its function of trimming MHC class I‐presented peptides in vivo and the role of this in immunodominance are also well recognized 13.

Other factors that may influence this outcome of divergent memory responses include TCR avidity of the responses, naïve precursor frequencies, and peptide stability. The discrete nature of the differences seen in the populations suggests a major difference in sustained peptide‐MHC availability rather than small quantitative differences that may impact on immunodominance. Finally, the MHC may be of relevance, where the Gp34 epitope is restricted by H‐2Kb, compared to that of the Gp33 epitope, which is restricted by H‐2Db 14.

Thus, the simplest explanation for our data, since we know that long‐term antigen availability drives memory inflation, is that the two different antigen‐specific T‐cell populations compete for peptide–MHC signals on APCs during the memory phase. This would fit with a model whereby peptides are cross‐presented on dendritic cells during priming, allowing presentation of many epitopes but directly presented during the memory phase, at which point competition for a more limited resource at the surface of a nonhematopoetic long‐lived cell may become critical. In other words, even if both epitopes are independently presented on different MHC molecules, the Gp34‐specific T cell pool can outcompete the Gp33 pool in vivo in the critical niche for antigen re‐encounter.

### Ad‐Gp and Ad‐Gp33 vectors generate a protective response

We then went on to look at whether these responses would provide protection against challenge. Mice immunized with the Ad‐Gp or the Ad‐Gp33 minigene vector received an LCMV challenge at day 50. Figure 1d shows the quantification of virus (measured by plaque assay) present in full‐length Ad‐Gp or Ad‐Gp33 minigene‐immunized mice in the spleen (i) and inguinal lymph nodes (ii) at day 4 after LCMV_WE_ challenge, compared to naïve controls. No virus could be detected in neither the Ad‐Gp nor the Ad‐Gp33 minigene immunized mice at this time‐point. The system demonstrates that minigene vectors can afford full protection, directly comparable to that of full‐length proteins. A number of studies have previously demonstrated protection, including those with the adjuvant effect of linkage to the invariant chain 15, 16 and those with full‐length gp inserts 17, 18. We had predicted that our full‐length Ad‐Gp vector would recapitulate this robust protection, and here, we show that the minimal Gp33 epitope was similarly sufficient.

### An Ad‐Gp34 minigene vector further enhances the inflationary response over the Ad‐Gp33 minigene

**Figure 2:**
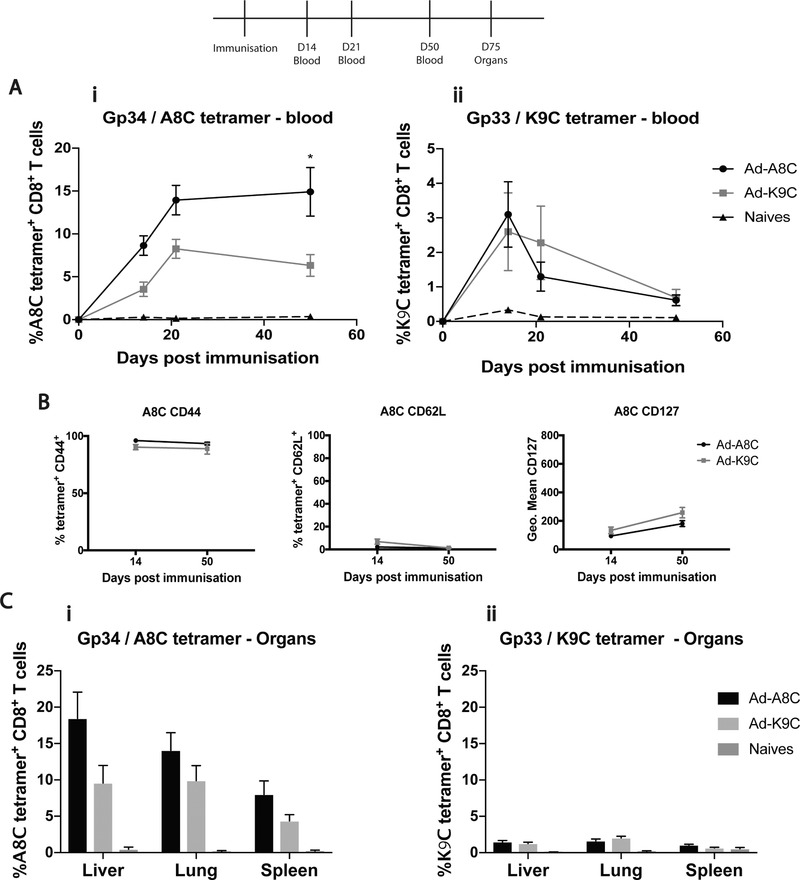
**(A)** Gp34/A8C **(i)** and Gp33/K9C **(ii)** tetramer‐specific responses by flow cytometry in blood at time‐points 14, 21, and 50 post‐immunization with Ad‐Gp34 compared to Ad‐Gp33 and naïve mice. Data pooled from two separate experiments: *n* = 12. Mean ± SEM shown. Unpaired *t*‐test with Welch's correction: *p ≤ 0.01. (**B)** A8C and K9C tetramer‐specific CD8^+^ T cells showing CD44, CD62L, and CD127 positivity by flow cytometry at day 21 and 50‐post immunization with Ad‐Gp34. Data shown from one experiment (*n* = 6), and representative of two independent experiments (*n* = 12). Mean ± SEM shown. (**C)** Gp34/A8C **(i)** and Gp33/K9C **(ii)** tetramer‐specific responses by flow cytometry in organs at day 75‐post immunization with Ad‐Gp34, compared to Ad‐Gp33 immunized and naïve mice. Data taken from two pooled experiments (*n* = 12). Mean ± SEM shown.

The Ad‐Gp34 minigene was later synthesized, to assess Gp34/A8C tetramer‐specific responses and to gauge the maximum level of response. The kinetic of inflationary cells in blood against the Gp34/A8C epitope revealed that inflation was significantly enhanced when compared to Ad‐Gp33 (Fig. 2a). Again, the inflationary kinetic was sustained, as demonstrated by both the phenotypic markers (Fig. 2b), enrichment within organs (Fig. 2c), and functionality (Supporting Information Fig. 2).

### An Ad‐disrupted D8V (LacZ) vector allows for inflation of I8V specific CD8^+^ T cell responses

**Figure 3:**
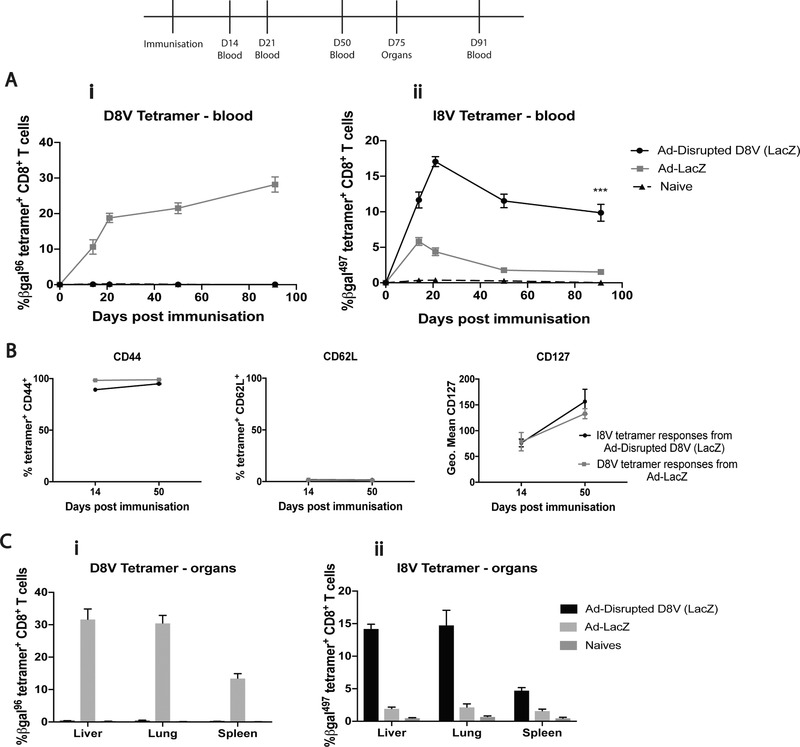
**(A)** β‐gal_96‐103_/D8V **(i)** and β‐gal_497‐504_/I8V **(ii)** tetramer‐specific responses by flow cytometry in blood at time‐points 14, 21, 50, and 91 post‐immunization with either Ad‐Disrupted D8V (LacZ) or Ad‐LacZ compared to naives. Data pooled from two separate experiments up to day 50: *n* = 12 and a single experiment at day 91: *n* = 6. Mean ± SEM shown. Unpaired *t*‐test with Welch's correction: ***p ≤ 0.0006. (**B)** I8V tetramer‐specific CD8^+^ T cells following Ad‐disrupted D8V (LacZ) and D8V tetramer‐specific CD8^+^ T cells following Ad‐LacZ immunization showing CD44, CD62L, and CD127 positivity by flow cytometry at day 21 and 50‐post immunization. Data shown from one experiment (*n* = 6), and representative of two independent experiments (*n* = 12). Mean ± SEM shown. (**C)** D8V **(i)** and I8V **(ii)** tetramer‐specific responses by flow cytometry in organs at day 75‐post immunization with Ad‐disrupted D8V (LacZ), compared to Ad‐LacZ immunized and naïve mice. Data from two pooled experiments (*n* = 12). Mean ± SEM shown.

Having shown the potential impact of competition between epitopes on inflation using the Gp33 and Gp34 minigene vectors, we were prompted to revisit the role of antigen context and competition in longer length inserts. We generated an Ad‐LacZ vector with the inflationary D8V epitope disrupted (nonfunctioning) through modification of an anchor residue. Figure 3a(i) shows the absence of D8V‐tetramer specific responses in the Ad‐Disrupted D8V (LacZ) vector, while Figure 3a(ii) shows the inflationary kinetic in blood from I8V‐tetramer specific responses from this same vector. The features of these cells are inflationary, demonstrated through the inflationary phenotype (Fig. 3b), high distribution in peripheral organs (Fig. 3c), and the functionality (Supporting Information Fig. 3). This striking result suggests that in the absence of inter‐epitope competition even naturally processed non‐inflationary epitopes can promote inflation.

### Concluding remarks

Overall, this work suggests a hierarchy of factors involved in deciding which epitopes are likely to drive inflation from within the context of the larger protein. These factors may include antigen context, processing (including immunoproteasome independency), binding affinity, MHC, and possibly further associated factors from the vector (adenoviral or MCMV) itself. Mutation of a single amino acid within a protein antigen can have a huge impact on the memory behavior of a T cell response to an entirely independent peptide. These data are of relevance to the development of adenoviral vectored vaccines, in consideration of the chosen peptide immunogen.

## Materials and methods

### Animals

Oxford, UK: Initial pilot experiments for the Ad‐Gp33 and Ad‐Gp vectors and all Ad‐Gp34 and Ad‐LacZ experiments were performed in Oxford according to UK Home Office regulations (project licence number 30/2744 and 30/3293). Mice (females aged 6 ± 2 weeks) were maintained in specific pathogen‐free (SPF) conditions in individually ventilated cages and fed on a normal chow diet. C57BL/6 mice were purchased from Harlan (UK).

St. Gallen, Switzerland: All challenge experiments and Ad‐Gp33/Ad‐Gp experiments were performed in St. Gallen according to the Swiss Federal and Cantonal guidelines (Tierschutzgesetz) under the permission SG10/14. Mice and conditions were matched to those for Oxford. C57BL/6 mice were purchased from Charles River (Switzerland).

### Adenoviral constructs

Replication‐deficient recombinant adenoviruses (AdHu5) expressing the full 1.5kb LCMV gp and Gp33‐41 (KAVYNFATC) epitope with an HCMV promoter (Ad‐Gp and Ad‐Gp33, respectively) were purchased from Vector BioLabs (Pennsylvania, USA).

Ad‐Gp34 (AVYNFATC), Ad‐LacZ (β‐gal/LacZ), and Ad‐Disrupted D8V (LacZ) were developed in Oxford, according to previously described techniques 9. The Ad‐disrupted D8V (LacZ) was designed with a sequence of DAPIATNV through the β‐gal_96‐103_, D8V (DAPIYTNV) section of the conventional β‐gal/LacZ.

All constructs were used intravenously (i.v.) at 1 × 10^8^ IU/mouse (all diluted into PBS at a volume of 200 µL per immunization/mouse) 9.

### Lymphocytic choriomeningitis virus

LCMV_WE_ (obtained from R.M. Zinkernagel) was propagated on mouse L929 fibroblast cells at a low MOI and was quantified as described 19. Mice were infected i.v. with 200 plaque‐forming units (pfu) of LCMV_WE_. Organs (spleens and inguinal lymph nodes) were then harvested at day 4‐post infection for plaque assays.

### LCMV plaque assays

LCMV plaque assays were performed using spleen and inguinal LN samples harvested from infected mice. Plaque assays were performed using permissive MC57 cell lines, and following methods as previously described 19, 20, 21.

### Peptides

The Gp33 (KAVYNFATC), Gp34 (AVYNFATC), D8V (DAPIYTNV) and I8V (ICPMYARV) peptides were purchased from Proimmune (Oxford, UK).

### Tetrameric MHC class I peptide complexes

MHC class I monomers (H‐2Db for Gp33, H‐2Kb for Gp34, I8V, and D8V) were kindly provided by the NIH Tetramer Core Facility, Emory University, USA: Gp33 and D8V tetramerized to APC and Gp34 and I8V tetramerized to PE. Cells were first incubated with the indicated tetramer at 37°C for 20 min.

### Antibodies

Cells were next incubated with the indicated antibodies (Supporting Information Table 1) at 4°C for 20 min.

### Flow cytometry

Blood or organs (liver, lung, and spleen) were prepared as previously described 6. Oxford, UK: Cells were acquired using a BD LSRII flow cytometer (Oxford, UK). St Gallen, Switzerland: Cells were acquired using a BD CANTO flow cytometer (Oxford, UK). Results were analyzed using Flowjo software (Tree star, USA) and according to EJI guidelines. The gating strategy is highlighted in Supporting Information Figure 4.

### Statistical analysis

All data is presented as the mean result from individual groups, with error bars indicating SEM. Statistical data analysis was performed using Graph‐Pad Prism version 7.0b for Mac (GraphPad Software, San Diego, CA, USA). Where indicated, statistical significance was calculated using an unpaired *t*‐test with Welch's correction.

## Conflicts of interest

The authors declare no commercial or financial conflict of interest.

**Abbreviations**
AdHu5: human adenovirus serotype 5
β‐gal: β‐galactosidase (also known as LacZ)
Gp: glycoprotein
LCMV: lymphocytic choriomeningitis virus
LMP7: low‐molecular mass protein‐7
MCMV: murine cytomegalovirus

## Supporting information

Supp. Table 1: Mouse antibody staining panels (with the clones indicated in brackets)
Supp. Figure 1: A) Gp33/K9C and Gp34/A8C tetramer‐specific responses measured by flow cytometry in blood at time‐points 21, 50, 75, 100 and 150 post‐immunisation with Ad‐Gp compared to naïve mice. Data is pooled from 3 separate experiments up to day 50: n = 10. Day 75 onwards is one experiment (n = 4). Mean ± SEM shown. B) A8C and K9C tetramer‐specific CD8+ T cells showing CD44, CD62L and CD127 positivity by flow cytometry at day 21 and 50‐post immunisation with Ad‐Gp. Data shown from 1 experiment (n = 6), and representative of 2 independent experiments. Mean ± SEM shown. C) A8C and K9C tetramer‐specific responses by flow cytometry in organs at day 75‐post immunisation with Ad‐Gp, compared to naïve mice. Data shown from 1 experiment (n = 4), and representative of 2 independent experiments. Mean ± SEM shown.
Supp. Figure 2: Intracellular cytokine staining measured by flow cytometry performed according to previously described protocols [6,9] showing IFN‐gamma and TNF‐alpha responses in splenocytes at day 75‐post immunisation with Ad‐Gp34/A8C compared to Ad‐Gp33/K9C immunised mice. Results demonstrate responses after stimulation with A8C/Gp34 and K9C/Gp33 peptide and compared to positive (PMA/Ionomycin) and negative (medium alone) controls. Data taken from 2 pooled experiments (n = 12). Mean ± SEM shown.
Supp. Figure 3: Intracellular cytokine staining measured by flow cytometry performed according to previously described protocols [6,9] showing IFN‐gamma and TNF‐alpha responses in splenocytes at day 75‐post immunisation with Ad‐Disrupted D8V (LacZ), compared to Ad‐LacZ immunised and naïve mice. Results demonstrate responses after stimulation with peptide and compared to positive (PMA/Ionomycin) and negative (medium alone) controls. Data taken from 1 experiment (n = 6). Mean ± SEM shown.
Supp. Figure 4: Overview of the flow cytometry gating strategy demonstrated (representative plots shown for each of the stages). Compensation controls were performed using UltraComp eBeads obtained from Invitrogen (USA). The gating strategy varied slightly dependent upon the platform: Click here for additional data file.
