# Supplementary material for: Divergent memory responses driven by adenoviral vectors are impacted by epitope competition
Source: Eur J Immunol. 2019 May 29;49(9):1356–63. doi: 10.1002/eji.201948143 (PMC6772135; doi:10.1002/eji.201948143)

European Journal of Immunology

Supporting Information
for
DOI 10.1002/eji.201948143

Julia M Colston, Claire Hutchings, Senthil Chinnakannan, Andrew Highton, Christian Perez-Shibayama, Burkhard Ludewig and Paul Klenerman

Divergent memory responses driven by adenoviral vectors are impacted by epitope competition

© 2019 The Authors. *European Journal of Immunology* published by WILEY-VCH Verlag GmbH & Co. KGaA, Weinheim.

Supplementary Data

Supp. Table 1: Mouse antibody staining panels (with the clones indicated in brackets):

**Oxford, UK:**	CD8a-eFluoro® 450 (53-6.7)	eBioscience (San Diego, USA)
	CD127-PE-Cy7 (A7R34)	
	CD62L-Alexa Fluor 700 (MEL-14)	BD Biosciences (Oxford, UK)
	CD44-FITC (IM7)	
	LIVE/DEAD Fixable Near-IR	Invitrogen (USA)
**St Gallen, CH:**	CD8a-PerCP (53-6.7)	Biolegend (San Diego, USA)
	CD127-PE-Cy7 (A7R34)	
	CD62L-APC-Cy7 (MEL-14)	
	CD44-FITC (IM7)	BD Biosciences (Oxford, UK)

Supp. Figure 1: A) Gp33/K9C and Gp34/A8C tetramer-specific responses measured by flow cytometry in blood at time-points 21, 50, 75, 100 and 150 post-immunisation with Ad-Gp compared to naïve mice. Data is pooled from 3 separate experiments up to day 50: n=10. Day 75 onwards is one experiment (n=4). Mean ± SEM shown. **B)** A8C and K9C tetramer-specific CD8$^+$ T cells showing CD44, CD62L and CD127 positivity by flow cytometry at day 21 and 50-post immunisation with Ad-Gp. Data shown from 1 experiment (n=6), and representative of 2 independent experiments. Mean ± SEM shown. **C)** A8C and K9C tetramer-specific responses by flow cytometry in organs at day 75-post immunisation with Ad-Gp, compared to naïve mice. Data shown from 1 experiment (n=4), and representative of 2 independent experiments. Mean ± SEM shown.

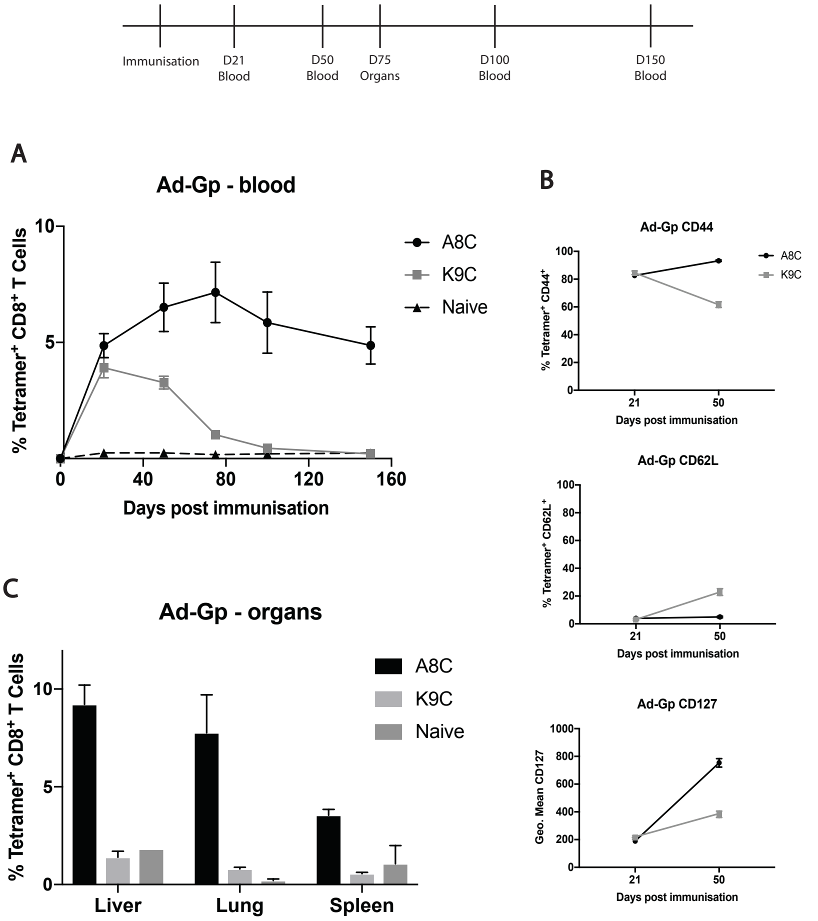

Supp. Figure 2: Intracellular cytokine staining measured by flow cytometry performed according to previously described protocols [6,9] showing IFN-gamma and TNF-alpha responses in splenocytes at day 75-post immunisation with Ad-Gp34/A8C compared to Ad-Gp33/K9C immunised mice. Results demonstrate responses after stimulation with A8C/Gp34 and K9C/Gp33 peptide and compared to positive (PMA/Ionomycin) and negative (medium alone) controls. Data taken from 2 pooled experiments (n=12). Mean ± SEM shown.

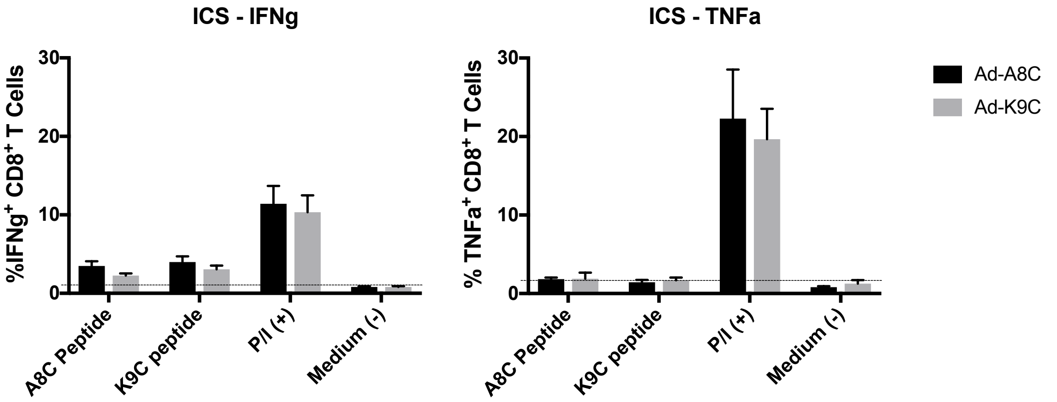

Supp. Figure 3: Intracellular cytokine staining measured by flow cytometry performed according to previously described protocols [6,9] showing IFN-gamma and TNF-alpha responses in splenocytes at day 75-post immunisation with Ad-Disrupted D8V (LacZ), compared to Ad-LacZ immunised and naïve mice. Results demonstrate responses after stimulation with peptide and compared to positive (PMA/Ionomycin) and negative (medium alone) controls. Data taken from 1 experiment (n=6). Mean ± SEM shown.

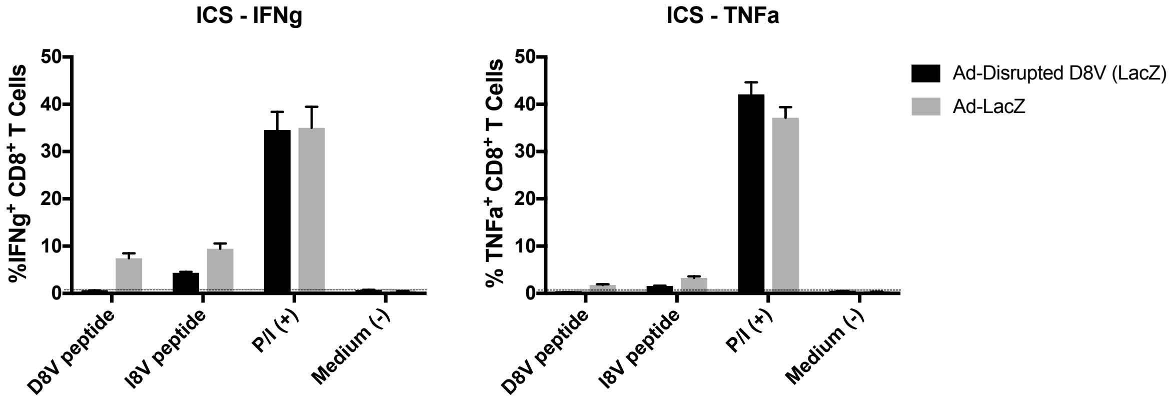

Supp. Figure 4: Overview of the flow cytometry gating strategy demonstrated (representative plots shown for each of the stages). Compensation controls were performed using UltraComp eBeads obtained from Invitrogen (USA). The gating strategy varied slightly dependent upon the platform:

- <u>BD LSRII (used for all Ad-A8C/Gp34 and Ad-Disrupted D8V experiments)</u>: lymphocytes > singlets (doublets excluded by gating on FSC-A x FSC-H) > Live/Dead > CD8$^+$ > tetramer positive > CD44/CD62L/CD127 positive (geometric mean for CD127),
- <u>BD CANTO (used for all Ad-Gp and Ad-K9C/Gp33 experiments)</u>: lymphocytes > CD8$^+$ > tetramer positive > CD44/CD62L/CD127 positive (geometric mean for CD127).

The middle panel shows the tetramer cloud for each of the tetramers (from left to right): D8V, I8V, A8C (Gp34) and K9C (Gp33).

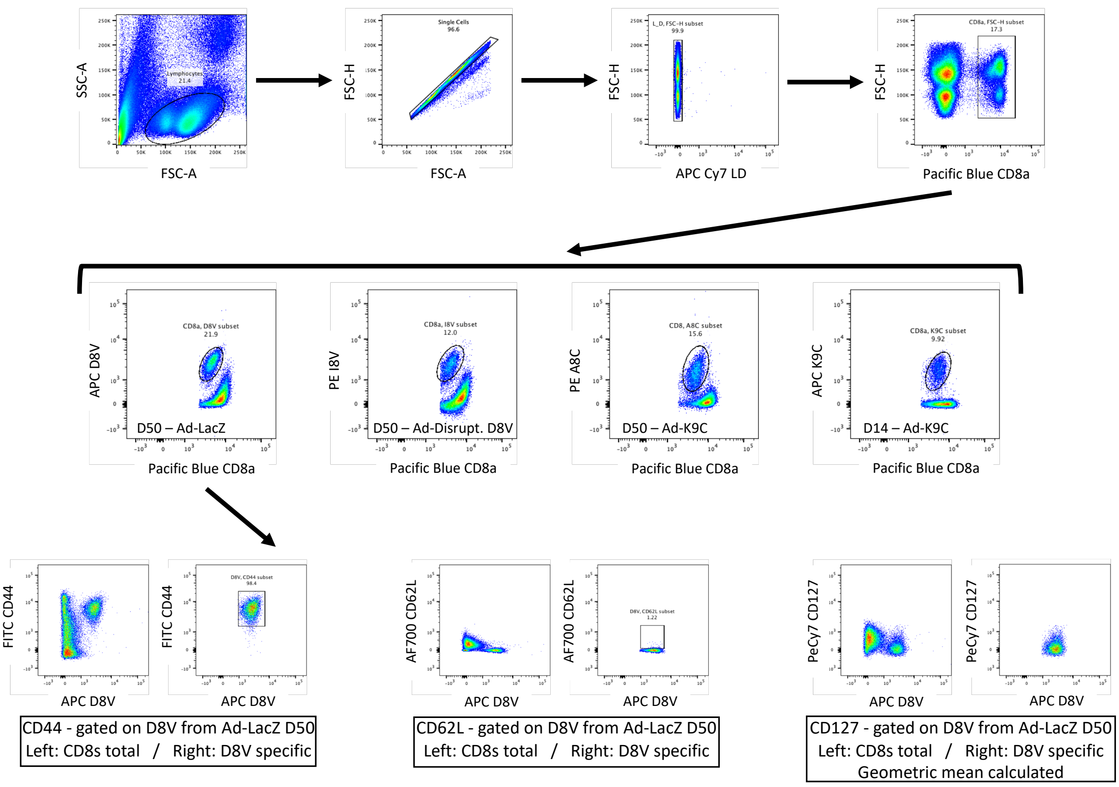

Supplement: Supplementary file 1 — Supp. Table 1: Mouse antibody staining panels (with the clones indicated in brackets) Supp. Figure 1: A) Gp33/K9C and Gp34/A8C tetramer‐specific responses measured by flow cytometry in blood at time‐points 21, 50, 75, 100 and 150 post‐immunisation with Ad‐Gp compared to naïve mice. Data is pooled from 3 separate experiments up to day 50: n = 10. Day 75 onwards is one experiment (n = 4). Mean ± SEM shown. B) A8C and K9C tetramer‐specific CD8+ T cells showing CD44, CD62L and CD127 positivity by flow cytometry at day 21 and 50‐post immunisation with Ad‐Gp. Data shown from 1 experiment (n = 6), and representative of 2 independent experiments. Mean ± SEM shown. C) A8C and K9C tetramer‐specific responses by flow cytometry in organs at day 75‐post immunisation with Ad‐Gp, compared to naïve mice. Data shown from 1 experiment (n = 4), and representative of 2 independent experiments. Mean ± SEM shown. Supp. Figure 2: Intracellular cytokine staining measured by flow cytometry performed according to previously described protocols [6,9] showing IFN‐gamma and TNF‐alpha responses in splenocytes at day 75‐post immunisation with Ad‐Gp34/A8C compared to Ad‐Gp33/K9C immunised mice. Results demonstrate responses after stimulation with A8C/Gp34 and K9C/Gp33 peptide and compared to positive (PMA/Ionomycin) and negative (medium alone) controls. Data taken from 2 pooled experiments (n = 12). Mean ± SEM shown. Supp. Figure 3: Intracellular cytokine staining measured by flow cytometry performed according to previously described protocols [6,9] showing IFN‐gamma and TNF‐alpha responses in splenocytes at day 75‐post immunisation with Ad‐Disrupted D8V (LacZ), compared to Ad‐LacZ immunised and naïve mice. Results demonstrate responses after stimulation with peptide and compared to positive (PMA/Ionomycin) and negative (medium alone) controls. Data taken from 1 experiment (n = 6). Mean ± SEM shown. Supp. Figure 4: Overview of the flow cytometry gating strategy demonstrated (representative plots s [file EJI-49-1356-s001.pdf]
